# Genetic evidence that advanced COVID-19 accelerates longitudinal brain atrophy: A Mendelian randomization study

**DOI:** 10.1097/MD.0000000000049310

**Published:** 2026-06-26

**Authors:** Jie Wen, Yuyao Chen, Jingwei Zhang, Zeming Tan, Zhiwei Xia, Sisi Yang, Hongwei Liu

**Affiliations:** a Department of Neurosurgery, Xiangya Hospital, Central South University, Changsha, Hunan, China; b Hypothalamic Pituitary Research Centre, Xiangya Hospital, Central South University, Changsha, Hunan, China; c National Clinical Research Center for Geriatric Disorders, Xiangya Hospital, Central South University, Changsha, Hunan, China; d Department of Epidemiology and Health Statistics, Xiangya School of Public Health, Central South University, Changsha, Hunan, China; e Department of Neurology, Hunan Aerospace Hospital, Hunan Normal University, Changsha, Hunan, China; f Department of Neurosurgery, Hunan Aerospace Hospital, Hunan Normal University, Changsha, Hunan, China.

**Keywords:** brain aging, COVID-19, genetic analysis, longitudinal MRI, mediation analysis, Mendelian randomization

## Abstract

Coronavirus disease 2019 (COVID-19) was reported to persist long-term in the brain and leave several long-term neurologic sequelae. However, the causal relationship between COVID-19 and brain aging is still unknown. The genome-wide association study (GWAS) data on COVID-19 phenotypes (susceptibility, hospitalization, and severity), involving a total of 5,779,391 participants, were collected from the COVID-19 Host Genetics Initiative. In addition, GWAS data on longitudinal changes in 15 brain structures, assessed via magnetic resonance imaging across the lifespan, were sourced from the ENIGMA Consortium and involved 15,640 participants. Two-sample Mendelian randomization was conducted to infer the causal relationship between COVID-19 and longitudinal brain changes. Multi-trait GWAS meta-analysis, colocalization, and fine-mapping analyses were performed to identify shared genetic etiologies. H3K27me3 ChIP-seq was used to evaluate the regulatory effect of colocalized loci. Two-step Mendelian randomization was applied to explore potential mediating mechanisms across multi-omics layers, including proteomics, metabolomics, and immunomics. Our results showed that COVID-19 hospitalization (β = −262.405, *P* = .041) and severity (β = −177.676, *P* = .049) were genetically associated with atrophied volume of total brain during longitudinal change. This suggests that individuals with advanced COVID-19 may be more susceptible to accelerated global brain aging. Caudate was genetically affected by all COVID-19 phenotypes. Seven variants were shared between advanced COVID-19 and global brain aging. rs117169628 was colocalized between advanced COVID-19 and global brain aging, and exerted an inhibitory effect on CDH15 expression, further strengthening the causality. Six metabolites, 1 protein, and 1 immune trait were identified as potential mediators. Our study indicates that advanced COVID-19 might be genetically associated with accelerated brain aging. Brain health should be paid more attention in long COVID-19.

## 1. Introduction

Coronavirus disease 2019 (COVID-19) has been a pandemic worldwide for over 3 years and contributed to substantial morbidity and mortality, especially for neurologic sequelae.^[1]^ A range of neurological complications, including cognitive impairment, anosmia, encephalopathy, cerebrovascular events, and persistent “brain fog,” have been documented during and after COVID-19 infection.^[2,3]^ The Global Burden of Disease study demonstrated that COVID-19-attributable neurological years lived with disability reached 30 to 40 years in adults.^[4]^ A population-based study revealed that the prevalence of cognitive impairment remained at 19.1% (95% CI = 11.5%–28.8%) among individuals with mild to severe severe acute respiratory syndrome coronavirus 2 infection 2 years postinfection.^[5]^ These disorders are structurally associated with whole-brain or region-specific atrophy. Observational studies reported that longitudinal magnetic resonance imaging (MRI) showed COVID-19 patients would have unrecovered atrophy for global brain and specific brain structures after long-term follow-up, which is a typical sign of brain aging.^[6–8]^ In addition, RNA-seq analysis of 54 postmortem brain samples revealed that the COVID-19 brain was enriched for aging pathways.^[9]^ In this study, we aimed to explore whether COVID-19 causally contributes to global and subregional brain atrophy from genetic analyses, as well as to uncover the potential underlying mechanisms.

Although observational studies could report the association, their inherent defect could not reveal causality, as confounders and reverse causation. Besides, brain aging is a relatively slow process that cannot be observed sufficiently quickly. Thus, the causal relationship between COVID-19 and brain aging is not clear and hard to establish. Mendelian randomization (MR) is a method to reveal the causality between exposures and outcomes by using single-nucleotide polymorphisms (SNPs) as unconfounded proxies, which mimics the randomized controlled trials (design because genetic variants are randomly assorted during meiosis. This approach could overcome inherent confounding bias and potential reverse causality, which commonly exists in observational research.^[10]^ Using MR, COVID-19 was found to be a risk factor for multiple neurodegenerative diseases.^[11]^

In this study, based on the genetic data from a large population, we used MR to investigate the effect of COVID-19 on global and regional aging for brain structures. Genetic analyses and two-step MR were performed to depict shared genetic etiology and potential mediating mechanisms. We found that advanced COVID-19 causally increased the risk of aging for the total brain.

## 2. Materials and methods

**Figure 1.**
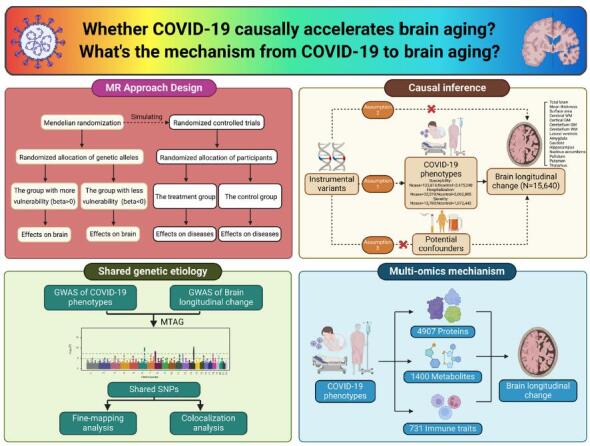
Flowchart of the study. Reprinted with permission from Biorender.com (2026).

The MR approach design and flowchart of this study are shown in Figure 1.

MR is a powerful analytical approach in genetic epidemiology that leverages genetic variants as instrumental variables (IVs) to investigate causal relationships between specific exposures and outcomes.^[10]^ MR operates by leveraging the random allocation of genetic variants that influence the exposure of interest, using these as genetic IVs in statistical analyses. This approach mimics the design of randomized controlled trials by taking advantage of genetic variation, which is fixed at conception and therefore not influenced by confounding factors that typically affect exposure-disease associations in traditional observational studies (Fig. 1). Thus, MR could provide a robust framework for establishing causal relationships between exposures and disease outcomes.

In this study, we used a two-sample MR analysis to identify the causal effects of COVID-19 risk (susceptibility, hospitalization, and severity) on brain aging (longitudinal MRI changes in brain structure across the lifespan) based on several genome-wide association study (GWAS) summary statistics. Then, multi-trait GWAS meta-analyses, colocalization, and fine-mapping analysis were performed to identify shared causal variants and shared genetic etiology between COVID-19 and brain aging. At last, two-step MR analyses were conducted to explore potential mechanisms from COVID-19 to brain aging from multi-omics, including proteomics, metabolomics, and immunomics.

### 2.1. Data sources for COVID-19 risk

Data for this article were obtained from publicly available GWAS datasets, and no individual-level data were available. Therefore, no ethical approval or informed consent was required. Summary statistics of COVID-19 risk were obtained from the latest version of COVID-19 host genetics GWAS meta-analyses released on April 8, 2022 (round 7, GRCh38, https://www.covid19hg.org/results/r7/).^[12]^ In total, 4,872,932 participants of European origin were included. The diagnosis of COVID-19 cases was based on laboratory-confirmed severe acute respiratory syndrome coronavirus 2 infection, electronic health records, or physician diagnosis of COVID-19, or self-reporting by patients with COVID-19 infection. Three phenotypes were downloaded from this GWAS: Confirmed COVID-19 patients versus the population controls, including 122,616 cases and 2,475,240 controls (COVID-19 susceptibility, HGI_C2_ALL_leave); confirmed COVID-19 hospitalized patients versus the population controls, including 32,519 cases and 2,062,805 controls (COVID-19 hospitalization, HGI_B2_ALL_leave); confirmed COVID-19 patients with very severe respiratory versus the population controls, including 13,769 cases and 1,072,442 controls (COVID-19 severity, HGI_A2_ALL_leave).

### 2.2. Data sources for the longitudinal changes in brain structure across the lifespan

The longitudinal changes in brain structure across the lifespan-related GWAS data were obtained from the ENIGMA meta-analysis consortium.^[13]^ The change rates of 15 brain structure traits were measured from 15,640 individuals of European descent across 40 longitudinal cohorts worldwide, using longitudinal MRI. This GWAS provides comprehensive genetic information on brain aging and neurodegenerative processes. The 15 brain traits include total brain, mean thickness, and surface area of the cortex, cerebral white matter, cortical gray matter, cerebellum gray matter and white matter, lateral ventricle, and key nuclei underlying the primary function, including amygdala, caudate, hippocampus, nucleus accumbens, pallidum, putamen, and thalamus.^[14]^ They are profoundly affected during the process of brain aging. Total brain longitudinal brain change was deemed the main result of this study.

### 2.3. MR analyses

MR analyses were performed following 3 principles^[15]^: IVs are strongly related to the corresponding exposures (*P* < 5 × 10^–8^); IVs are independent of outcome and can only affect the outcome through exposure; and IVs are independent of potential confounders (alcohol, smoking, and BMI). SNPs clumped using the PLINK algorithm (LD < 0.001 and <1 MB from the index variant). *F*-statistics were calculated for each IV in the exposures using the formula (*R*^2^/*K*)/([1 − *R*^2^] [N − *K* − 1]), where *K* represents the number of SNPs and N is the sample size. The variance explained by SNPs was calculated using the formula 2 × EAF × (1 − EAF) × (Β/SE)^2^. IVs with *F* < 10 were excluded to eliminate weak instruments.

To eliminate the potential heterogeneity and the pleiotropy effects of IVs, several MR methods were used to identify the causal effects of COVID-19 risk and longitudinal MRI changes in brain structure in this paper. Random-effect inverse-variance weighted was performed for the main analysis, which combined the Wald ratios of the causal effect of each SNP on the outcome and provided the most accurate estimate.^[16,17]^ In addition, to provide more reliable estimates over a broader range, other MR analytical methods, including MR-Egger regression and weighted median methods, were performed as supplements to the inverse-variance weighted. The MR-Egger method only needs to satisfy the assumption that the direct effects of IVs and outcomes are independent of the effects of IVs associated with exposure factors. The MR-Egger algorithm can measure the average multiplicity of effects among IVs using intercept terms when there is a multiplicity of effects of IVs. Therefore, the hypothesis of IVs can be effectively assessed by the MR-Egger intercept term.^[18]^ The weighted median method provided consistent effect estimates that accurately calculate causal association effects even when <50% of genetic variation violates MR core assumptions.^[17]^ Cochran *Q* test and MR-Egger intercept test were further used to identify the presence of pleiotropy and assess the reliability of the results. A funnel plot was used to assess possible directional pleiotropy. A leave-one-out test was performed to identify peculiar SNPs and the robustness of the MR results.

To estimate the potential reverse causality, reverse MR was performed. The significance threshold for longitudinal brain changes was set at *P* < 1 × 10^−5^, which was considered suggestive but did not reach the genome-wide significance level of 5 × 10^−8^ for SNPs.

MR estimates are expressed as β values and standard errors, which provide the risk coefficient of COVID-19 risk for longitudinal changes in brain structure across the lifespan. All analyses were performed using R (version 4.0.5) packages (TwoSampleMR, version 0.5.6).

### 2.4. Multi-trait analysis of GWAS

To identify risk SNPs associated with the composite phenotype encompassing COVID-19 and brain longitudinal change, we performed a cross-trait meta-analysis using the multi-trait analysis of GWAS (MTAG) method with GWAS summary statistics.^[19]^ The MTAG approach assumes equal SNP heritabilities across traits and consistent genetic covariances between them. To ensure the selection of independent SNPs, we applied PLINK clumping with parameters set to *r*^2^ = 0.05 and a distance threshold of 1000 kb. Subsequently, independent shared SNPs not predominantly driven by a single trait, defined as 5 × 10^−8^ < *P* < .05 in both traits, were identified as novel shared SNPs.^[20]^

### 2.5. Colocalization analyses

We performed a colocalization analysis using the Coloc tool.^[21]^ Summary statistics were extracted for variants located within 500 kb of the index SNP at each shared locus. Posterior probabilities were calculated for H4 (PP.H4, representing the probability that both traits share a single causal variant) and H3 (PP.H3, indicating the likelihood that each trait is associated with different causal variants). A locus was considered colocalized if PP.H4 exceeded 0.8.

### 2.6. Fine-mapping credible set analysis

To refine the identification of causal variants, we constructed a 99% credible set using an efficient Bayesian fine-mapping approach, FM-summary, as described in https://github.com/hailianghuang/FM-summary. For this analysis, we selected variants located within a 500 kb radius of each significant index SNP identified in the cross-trait meta-analysis and used them as input for FM-summary. This method calculates the posterior inclusion probability for each variant, representing the likelihood of a true association with the trait or disease. To define the 99% credible set, SNPs were ranked in descending order of posterior inclusion probability, and their probabilities were cumulatively summed until reaching at least 99%. Additional methodological details can be found in previous reports.^[22]^

### 2.7. Regulatory role of cis-acting quantitative trait loci

In this study, we queried colocalized loci to evaluate supporting evidence for whether these variants act as regulatory variants. We utilized the H3K27me3 ChIP-seq (an inhibitory regulatory marker) dataset from the human brain, which was collected from ChIP-atlas (number: SRX16495378).^[23]^ For visualization, we downloaded BigWig files and input them into the IGV software (version 2.16.2).^[24]^

### 2.8. Two‐step MR

A two-step MR analysis was conducted to explore potential mediators in the causal pathway from COVID-19 to brain aging. Summary-level GWAS data for the plasma proteome (4907 proteins), metabolomics (1400 metabolic traits), and immunomics (731 immune traits) were retrieved from 35,559,^[25]^ 8299,^[26]^ and 3757^[27]^ Europeans, respectively.

In the first step of the two-step MR analysis, multi-omics candidates of mediators were conducted (*P* < 1 × 10^−5^, clump_kb = 10,000, and *r*^2^ = 0.001) as exposures to identify potential proteins causally associated with the risk of brain aging (as outcomes). In the second step, the effects of COVID-19 phenotypes (as exposures) on these identified risk candidates (as outcomes) were evaluated using MR. The mediating proportion was calculated as β1 (the effect of the mediator on brain aging) × β2 (the effect of COVID-19 on the mediator)/β_total_ (the effect of COVID-19 on brain aging).^[28]^

## 3. Results

### 3.1. Genetically causal effects of COVID-19 phenotypes on brain aging

In total, 14, 30, and 27 SNPs were identified as IVs for COVID-19 susceptibility, hospitalization, and severity, respectively (Table S1, Supplemental Digital Content). All the *F* values for IVs were above 10, indicating the strength of the proxies (Table S1, Supplemental Digital Content).

**Figure 2.**
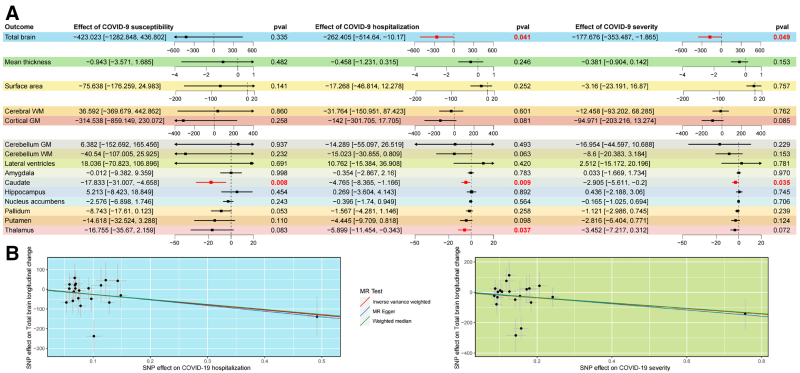
Two-sample Mendelian randomization results. (A) Genetic effects of COVID-19 phenotypes on 15 brain longitudinal changes. (B) Scatter plots for the genetic effect of COVID-19 hospitalization (left) and severity (right) on total brain aging. COVID-19 = coronavirus disease 2019.

Two-sample MR results showed that COVID-19 hospitalization (β = −262.405, *P* = .041) and severity (β = −177.676, *P* = .049) were genetically associated with atrophied volume of total brain during longitudinal change (Fig. 2A and Table S2, Supplemental Digital Content). This suggested that advanced COVID-19 patients might be more vulnerable to accelerated global brain aging.

Scatter plots (Fig. 2B) and leave-one-out (Fig. S1, Supplemental Digital Content) plots showed the robustness across different MR methods, no peculiar SNP, and no pleiotropy. Pleiotropy tests by the MR-Egger intercept test and heterogeneity tests by the Cochran *Q* test showed no significant pleiotropy and heterogeneity for these main MR results (Tables S3 and S4, Supplemental Digital Content). Reverse MR showed that total brain longitudinal change exerted no significant effects on COVID-19 phenotypes (*P* > .05; Table S5, Supplemental Digital Content). These sensitivity analyses showed the robustness of the main MR results.

Besides, for the brain subregion, caudate was affected by COVID-19 susceptibility (β = −17.833, *P* = .008), hospitalization (β = −4.765, *P* = .009), and severity (β = −2.905, *P* = .035), with all effects indicating a tendency toward atrophy. This suggested that the caudate may be a region more vulnerable to aging influenced by COVID-19.

### 3.2. Identification of shared genetic etiology and colocalization between advanced COVID-19 and total brain aging

**Figure 3.**
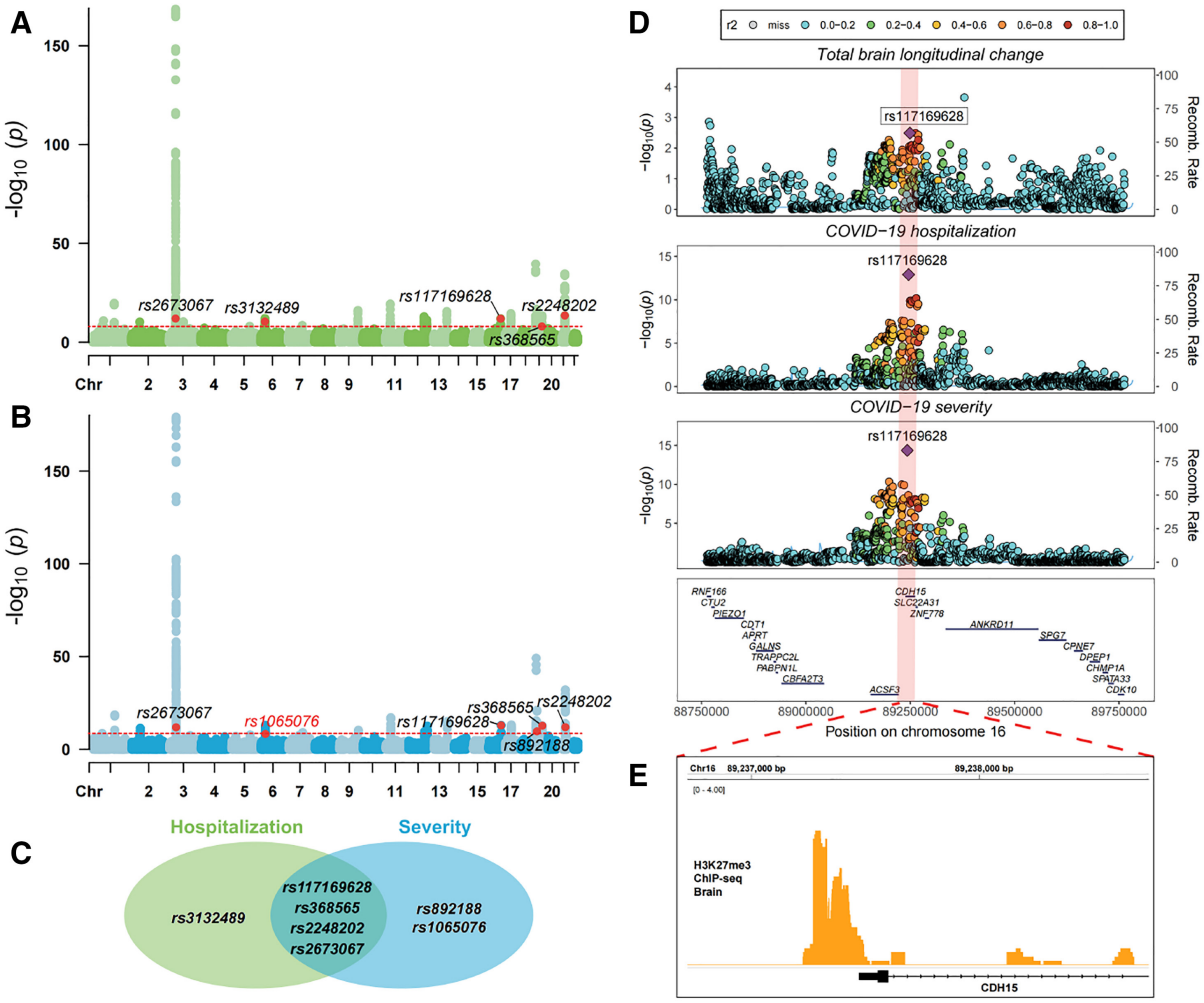
Share genetic etiology between advanced COVID-19 and total brain aging. (A) Multi-trait analysis of GWAS (MTAG) results between COVID-19 hospitalization and total brain longitudinal change. (B) MTAG results between COVID-19 severity and total brain longitudinal change. (C) Venn plots for the overlapped shared SNPs of MTAG results. (D) Colocalization analysis. (E) Peak visualization of H3K27me3 ChIP-seq in the CDH15 region. COVID-19 = coronavirus disease 2019, GWAS = genome-wide association study, MTAG = multi-trait analysis of GWAS, SNPs = single-nucleotide polymorphisms.

To investigate the shared genetic etiology between advanced COVID-19 (hospitalization and severity) and total brain aging, we performed MTAG to find shared risk SNPs. Five independent SNPs were identified as shared loci between COVID-19 hospitalization and total brain aging (Fig. 3A), and 6 independent SNPs were identified as shared loci between COVID-19 severity and total brain aging (Fig. 3B). Among these, rs1065076 was identified as a novel shared locus. Furthermore, 4 SNPs (rs117169628, rs2248202, rs368565, and rs2673067) overlapped between the shared loci of COVID-19 hospitalization and total brain aging and COVID-19 severity and total brain aging, suggesting a similar genetic etiology and highlighting the core roles of these 4 SNPs underlying advanced COVID-19 effects (Fig. 3C).

rs117169628 was colocalized for both pairs of COVID-19 hospitalization and total brain aging (PP.H4 = 0.99) and COVID-19 severity and total brain aging (PP.H4 = 0.99; Fig. 3D), which further strength the causal evidence. rs117169628 was found to be located at the promoter region of the *CDH15* gene, as revealed by H3K27me3 ChIP-seq (Fig. 3E). This suggests that rs117169628 may exert potential inhibitory regulation on the expression of CDH15. Besides, rs3132489 was colocalized for COVID-19 hospitalization and total brain aging (PP.H4 = 0.85). rs368565 was colocalized for COVID-19 severity and total brain aging (PP.H4 = 0.83). For each significant locus identified through MTAG, a 99% credible set of causal SNPs was determined using FM-summary for further analysis; details are provided in Tables S7 and S8, Supplemental Digital Content.

### 3.3. The potential mediators and underlying mechanisms from advanced COVID-19 to total brain aging

**Figure 4.**
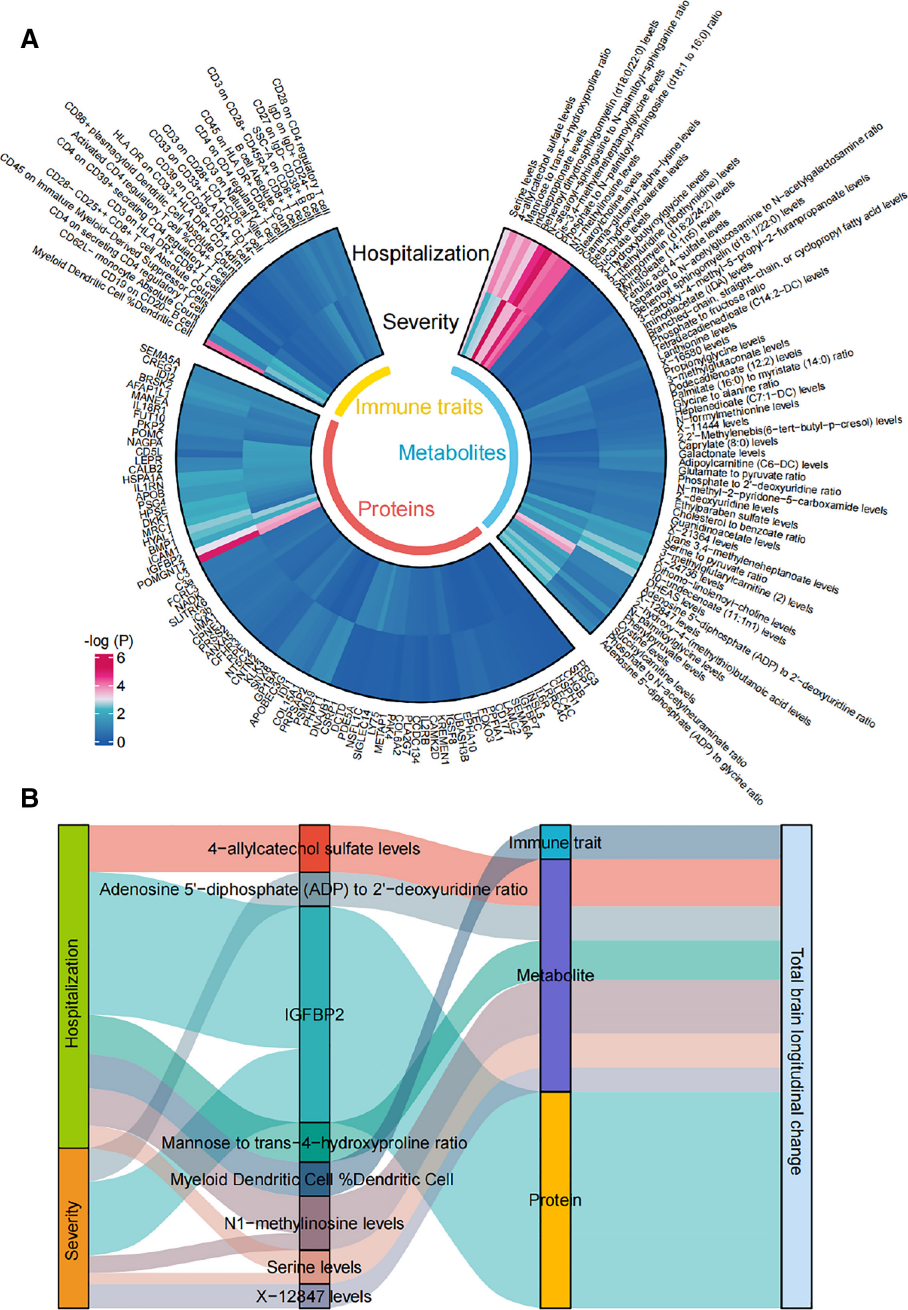
Two-step MR results. (A) Genetic effects of advanced COVID-19 on total brain longitudinal change. (B) Sanky plot for the mediators and mediating proportion from advanced COVID-19 to total brain aging. The width of the stripe represented the relative mediating proportion. COVID-19 = coronavirus disease 2019, MR = Mendelian randomization.

In the first step of two-step MR, we identified 83 proteins, 63 metabolites, and 23 immune traits associated with total brain aging, which were deemed as candidate mediators (Fig. 4A). In the second step, COVID-19 hospitalization and severity were significantly associated with 6 and 5 candidate mediators, respectively (Fig. 4A, B and Table S9, Supplemental Digital Content). The mediating proportion for these mediators ranged from 5.74% to 73.47% (Table S9, Supplemental Digital Content). IGFBP2, N1–methylinosine levels, and serine levels were the overlapped mediators between COVID-19 hospitalization and severity, suggesting a similar potential mechanism underlying advanced COVID-19 to brain aging.

## 4. Discussion

As far as we know, this is the first comprehensive MR study to investigate whether COVID-19 causally induces brain aging. Our findings demonstrated that advanced COVID-19, including hospitalization and severity, was genetically associated with reduced total brain volume over longitudinal changes – essentially indicative of accelerated brain aging. These results suggested that patients with advanced COVID-19 may be more vulnerable to this process of accelerated brain aging. Besides, we identified the shared genetic etiology and core causal variants between advanced COVID-19 and total brain aging. At last, we uncovered mediators from advanced COVID-19 to total brain aging at multi-omics. Our study provided a novel understanding of the COVID-19 effects on the brain and emphasized the potential neurologic sequelae for COVID-19 patients.

Notably, the caudate nucleus demonstrated a significant association with advanced COVID-19 across all 3 phenotypes, representing one of our most consistent and biologically important findings. This subregion – critical for motor control, cognition, and frontostriatal circuitry – is frequently affected in post-COVID patients. Both clinical and preclinical studies have reported COVID-related alterations in basal ganglia structures, including atrophy, metabolic dysfunction, and microglial activation,^[8,29]^ aligning with our genetic causal evidence. These findings highlight the importance of caudate nucleus detection and protection in the post-COVID era.

rs117169628 was identified as a key colocalized variant between advanced COVID-19 and total brain aging. Situated near CDH15 – which encodes a cell-adhesion molecule critical for synaptic formation, plasticity, and neuronal connectivity – this variant highlights a pathway fundamental to brain structure and function. CDH15 dysfunction has been linked to cognitive disabilities,^[30]^ a hallmark of brain aging. These CDH15-mediated processes are vulnerable to neuroinflammation and viral disruption, consistent with observations from post-COVID brains.^[31,32]^ Integrating these lines of evidence suggests that CDH15-related mechanisms may mechanistically contribute to brain atrophy in advanced COVID-19. Our findings indicate that patients with advanced COVID-19 may experience accelerated brain aging through rs117169628-mediated regulation of CDH15 levels.

IGFBP2 was identified as the mediator with the highest mediating proportion in the pathway from advanced COVID-19 to brain aging. Previous research has shown that IGFBP2 levels are elevated in the plasma of patients with severe COVID-19 compared to those with moderate COVID-19 and healthy controls.^[33]^ In the brain, IGFBP2 levels have been associated with atrophy in multiple brain regions and the progression of Alzheimer’s disease.^[34]^ These findings further support our findings. Targeting such mediators could potentially prevent or mitigate accelerated brain aging.

Previous studies have reported long-term neurologic sequelae after recovering from COVID-19. In a large study with 236,379 survivors of COVID-19, 34% were diagnosed with neurologic or psychiatric disorders after their COVID-19 diagnosis 6 months later.^[2]^ As COVID-19 remains a global pandemic, a significant proportion of the human population is likely to be affected by this infectious disease. The rapid evolution of the virus may further increase the likelihood of reinfections. From a public health perspective, our findings underscore the importance of monitoring brain aging following COVID-19 infection. Proactive measures, such as vaccination, early antiviral treatments, and neuroprotective therapies, could be pivotal strategies to mitigate the negative effects of COVID-19 on brain health.

However, this study has some limitations. First, MR results could be affected by the underlying pleiotropy, where a single genetic variant influences multiple traits. Second, the GWASs included in this study were only collected from individuals of European descent, which limits the generalizability of the findings to the entire population. Third, this study primarily relied on genetic analyses; further real-world population investigations and laboratory explorations are warranted in the future. Lastly, in this exploratory study, to give more clues and suggestions, we did not correct for multiple testing to find more potential risks for COVID-19-brain associations, as in previous studies.^[35–37]^ Thus, possible false positive results could not be excluded.

## 5. Conclusion

This first large-scale MR reveals the causal relationship between COVID-19 and accelerated brain aging. We found that advanced COVID-19 is genetically associated with accelerated global brain aging. Positive prevention and therapy for COVID-19 might be worthwhile for keeping the brain healthy. The biological mechanisms behind this association should be further investigated in the lab.

Supplemental Digital Content “Table S6” is available for this article.

## Acknowledgments

The authors express gratitude to the public databases, websites, and software used in the paper. The authors are grateful to the High Performance Computing Center of Central South University for partial support of this work.

## Author contributions

**Methodology:** Jie Wen, Zeming Tan, Hongwei Liu.

**Project administration:** Jie Wen, Hongwei Liu.

**Validation:** Jie Wen.

**Visualization:** Jie Wen.

**Writing – original draft:** Jie Wen, Jingwei Zhang, Hongwei Liu.

**Writing – review & editing:** Jie Wen, Yuyao Chen, Jingwei Zhang, Zeming Tan, Zhiwei Xia, Sisi Yang, Hongwei Liu.

**Supervision:** Yuyao Chen, Zhiwei Xia, Sisi Yang, Hongwei Liu.

**Conceptualization:** Jingwei Zhang, Zhiwei Xia, Hongwei Liu.

**Investigation:** Jingwei Zhang, Hongwei Liu.

**Funding acquisition:** Jie Wen, Hongwei Liu.

**Data curation:** Jie Wen, Jingwei Zhang, Hongwei Liu.

**Resources:** Jie Wen, Jingwei Zhang, Hongwei Liu.




















