# Supplementary figures and images for: Genetic evidence that advanced COVID-19 accelerates longitudinal brain atrophy: A Mendelian randomization study
Source: Medicine (Baltimore). 2026 Jun 26;105(26):e49310. doi: 10.1097/MD.0000000000049310 (PMC13313741; doi:10.1097/MD.0000000000049310)

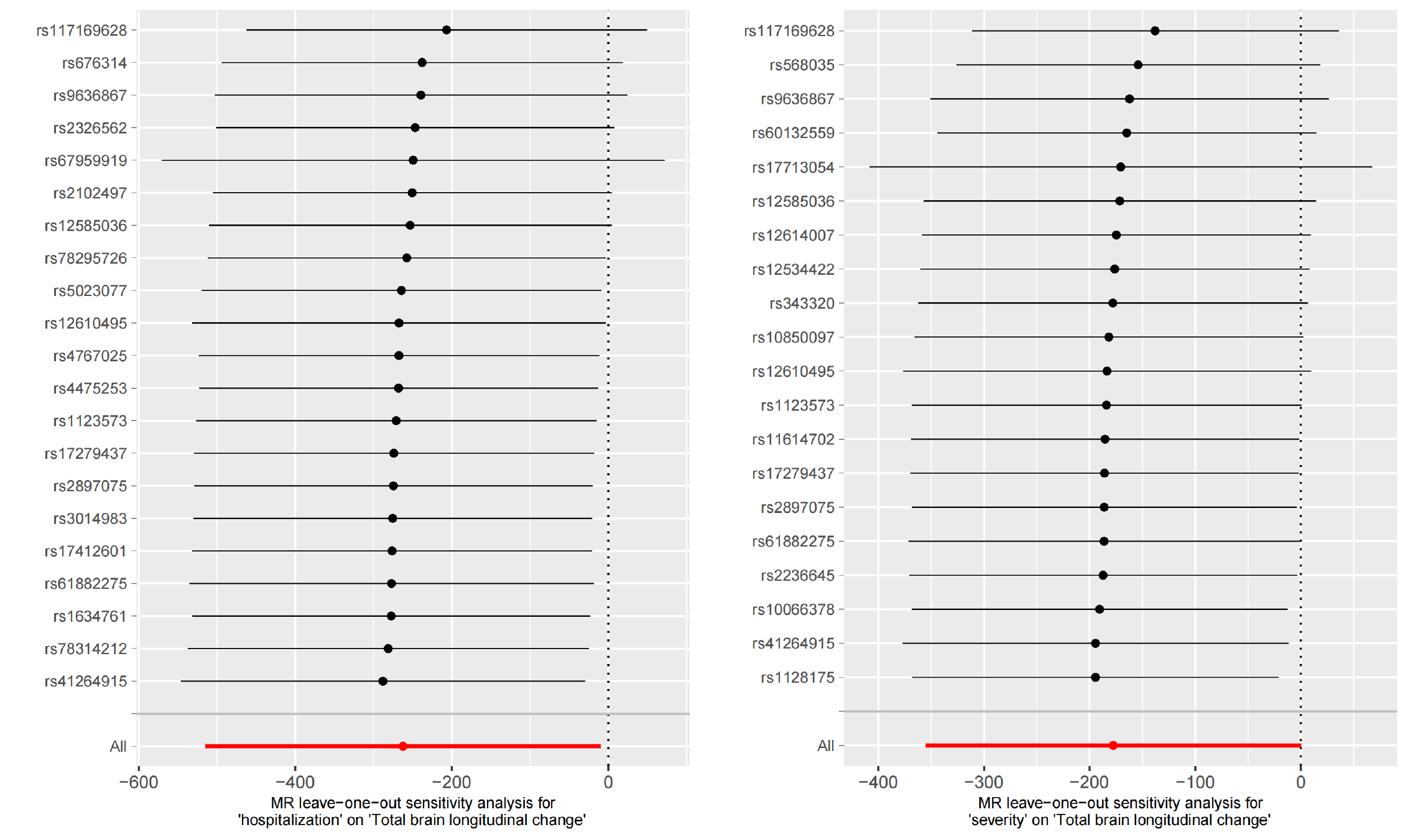

Supplement: Supplementary file 3 [file medi-105-e49310-s003.tif]
